# Disordered eating behaviors among female undergraduates in Sichuan, China: key associated factors from a province-wide survey

**DOI:** 10.3389/fpsyg.2026.1679944

**Published:** 2026-02-17

**Authors:** Danli He, Ching Sin Siau, Hui Chin Koo, Choy Qing Cham, Masoumeh Alavi, Agnes Shu Sze Chong, Harvinder Kaur Gilcharan Singh

**Affiliations:** 1 Centre for Health Education and Counselling, School of Foreign Languages, Yibin University, Yibin, China; 2 Centre for Community Health Studies, Faculty of Health Sciences, Universiti Kebangsaan Malaysia, Kuala Lumpur, Malaysia; 3 Faculty of Applied Sciences, Tunku Abdul Rahman University of Management and Technology, Kuala Lumpur, Malaysia; 4 School of Applied Psychology, Griffith University, Brisbane, QLD, Australia

**Keywords:** body image flexibility, body dissatisfaction, disordered eating behaviors, fatness concern, female undergraduates, perceived sociocultural pressures

## Abstract

**Background:**

Female undergraduates are at elevated risk for developing eating disorder symptoms. This cross-sectional study examined predictors of three disordered eating behaviors—uncontrolled eating, emotional eating, and cognitive restraint—among Chinese female undergraduates.

**Methods:**

A total of 1,727 participants (mean age = 19.73 ± 1.47 years) completed a web-based survey assessing demographics, sociocultural pressures, body dissatisfaction, fatness concern, body image flexibility, and disordered eating behaviors. Binary logistic regression was used to identify predictors.

**Results:**

Over half of the participants reported high levels of disordered eating behaviors. Uncontrolled eating was significantly associated with older age, higher perceived sociocultural pressures, lower body image flexibility, studying at China West Normal University, and fatness concern. Emotional eating was associated with higher perceived sociocultural pressures, body dissatisfaction and fatness concern. Cognitive restraint was associated with higher perceived sociocultural pressures, fatness concern, and body dissatisfaction. Participants who saw themselves as too fat had higher odds of showing emotional eating and cognitive restraint, while those who felt too thin had lower odds of showing cognitive restraint.

**Conclusion:**

Perceived sociocultural pressures, body dissatisfaction, and weight-related concerns were key predictors of disordered eating behaviors, highlighting the need for multifaceted interventions tailored to these psychological and sociocultural factors.

## 1 Introduction

Disordered eating behaviors (DEBs) are prevalent across both Eastern and Western contexts and represent a significant public health concern (Alhaj et al., 2022). Although DEBs do not necessarily meet diagnostic criteria for clinical eating disorders (EDs), accumulating evidence suggests that DEBs lie on an eating-pathology continuum and often precede ED onset, increasing the risk of developing full-threshold disorders (Uchôa et al., 2019; Unikel Santoncini et al., 2018). Recent global burden work indicates that ED burden may be substantially underestimated in population-level assessments. Specifically, because Global Burden of Disease (GBD) 2019 primarily captured anorexia nervosa and bulimia nervosa, an additional 41.9 million cases in 2019—largely attributable to binge-eating disorder and other specified feeding or eating disorder —were not represented, yielding an estimated total of approximately 55.5 million affected individuals worldwide (Santomauro et al., 2021; Vos et al., 2020). Consistent with these concerns, a systematic review reported that the global prevalence of EDs increased from 3.5 to 7.8% between 2000 and 2018, with markedly higher prevalence among females than males (Galmiche et al., 2019). In highly populous countries, even modest prevalence can translate into a substantial absolute burden; for example, more than 1.5 million people in China were estimated to be affected in 2019 (Li et al., 2021). In the United States, EDs also impose considerable societal and economic costs, underscoring their public health significance (Streatfeild et al., 2021). Given that EDs are associated with elevated mortality and long-term impairment, focusing on DEBs is critical because these subclinical behaviors may persist and intensify over time if left unaddressed (Alhaj et al., 2022; Deloitte Access Economics, 2020; Wang et al., 2019).

University life, particularly for female students, is a developmental period during which eating-related concerns and maladaptive behaviors may be heightened due to multiple transitions and stressors (Hao et al., 2022; Stok et al., 2018; Zhao et al., 2024). The transition to university is often accompanied by increased autonomy, changes in the social environment, peer comparison amplified by social media, and academic pressures, all of which can exacerbate mental health difficulties and behavioral problems, including DEBs (Aparicio-Martinez et al., 2019; Pacanowski et al., 2024; Stok et al., 2018). During this period, young adults—especially females—may be particularly vulnerable to body image concerns, which are closely linked to subclinical eating pathology (Gonidakis et al., 2018; Grogan, 1999; Zhao et al., 2024). Consistent with this, DEBs are commonly observed among university students. Behaviors such as frequent dieting, persistent preoccupation with food, weight, and body shape, as well as guilt and shame surrounding eating, may place students at elevated risk for progression toward clinical EDs (Coakley, 2022). Given that sociocultural appearance ideals and social media exposure are increasingly salient in many countries, it is important to examine DEBs within specific cultural contexts, including China, where rapid social change may intensify appearance-related pressures.

In China, disordered eating and related symptomatology appear to be common among university students. A meta-analysis reported that the prevalence of screening-identified ED symptoms among Chinese university students ranged from 1.96 to 39.8% between 2002 and 2021, with higher rates among females (Liu et al., 2022). At the policy level, China has emphasized student health promotion in higher education—for example, through mandatory physical education and the establishment of mental health education systems in universities (National Health Commission of the People’s Republic of China, 2016). However, despite these broader institutional efforts, the psychological mechanisms underlying DEBs remain insufficiently understood, particularly regarding sociocultural pressures, body dissatisfaction (BD), and body image flexibility.

### 1.1 Theoretical framework and integration of constructs

This study adopts a theoretically integrated approach combining Sociocultural Theory (Cash and Pruzinsky, 1990) and Acceptance and Commitment Therapy (ACT; Hayes et al., 1999) to explore the associative relationships between various factors and DEBs. Sociocultural Theory posits that social and cultural contexts influence individual behaviors and attitudes, particularly through the impact of media, family, and peers (Thompson et al., 1999). In collectivist societies like China, societal pressure to conform to communal ideals of beauty, often associated with a slim physique, leads to BD, which in turn contributes to unhealthy eating behaviors such as binge eating and restrictive dieting (Kakar et al., 2023; Liang et al., 2023; Tan et al., 2022; Wang et al., 2025).

Numerous studies have provided strong evidence that BD is a significant predictor of DEBs (Deek et al., 2024; Foster et al., 2024; Kapoor et al., 2022). BD, often manifesting as dissatisfaction with one’s weight, shape, or appearance, is a critical factor contributing to the onset of DEBs (Coelho et al., 2023; Yi et al., 2025; Zaccagni and Gualdi-Russo, 2023). Previous research has highlighted the significant influence of media, family, and peers on body image and eating behaviors. The widespread use of social media platforms such as TikTok, YouTube, and Instagram has intensified exposure to idealized body standards, leading to increased BD and a higher risk of DEBs, particularly among adolescents and young adults (Bonfanti et al., 2025; Güneş and Demirer, 2025; Suhag and Rauniyar, 2024; Tayhan Kartal and Yabancı Ayhan, 2021). Additionally, family interactions, such as the modeling of dieting behaviors by parents or negative body-related discussions, can significantly impact body image and eating behaviors. Research shows that adolescents whose parents engage in dieting are more likely to exhibit DEBs (Kontele et al., 2023; Lawrence et al., 2023; White et al., 2023). Peer influence also plays a critical role, with studies indicating that peer pressure and social comparisons, especially in university settings, can lead individuals to alter their eating behaviors to conform to societal beauty ideals, with peer teasing correlating with restrictive dieting (Wang et al., 2024).

A key concept in understanding how individuals cope with these pressures is body image flexibility, which refers to the capacity to accept and engage with body-related experiences—such as thoughts, emotions, and sensations—without letting them interfere with one’s values and actions (Sandoz et al., 2013). Body image flexibility allows individuals to experience BD or negative body image thoughts without being consumed by them or allowing them to dictate unhealthy behaviors, such as extreme dieting or binge eating (Linardon et al., 2021). Higher body image flexibility is associated with better psychological well-being, reduced body image distress, and fewer DEBs (Brichacek et al., 2023; Linardon et al., 2021), and plays a crucial role in helping individuals cope with sociocultural pressures and BD, leading to more adaptive eating behaviors.

Previous research has also shown that BMI, age, and fatness concern influence DEBs (Al-Shoaibi et al., 2024; Lehto et al., 2024; Liu et al., 2024; Solmi et al., 2022). This study aims to explore the factors (including demographic factors, perceived sociocultural pressures, BD, fatness concern, and body image flexibility) that are associated with three types of DEBs—uncontrolled eating (UE), emotional eating (EE), and cognitive restraint (CR)—as measured by the Three-Factor Eating Questionnaire. By investigating the relationships between these factors and DEBs, this study seeks to provide a deeper understanding of the psychological and sociocultural factors underlying DEBs among Chinese female university students.

## 2 Materials and methods

### 2.1 Study design and participants

This was a cross-sectional study conducted across five universities/colleges in Sichuan Province, China, from January to February 2024. To ensure regional representation and account for socioeconomic differences, institutions were first stratified by geographic region—Eastern, Western, Northern, and Southern Sichuan. One institution was randomly selected from each region using Microsoft Excel’s random number generator. Given the high concentration of universities in Western Sichuan, particularly in Chengdu, an additional institution from that region was included. The final sample comprised five institutions: Sichuan Academy of Tourism, Chengdu College of Arts and Sciences, Southwest University of Science and Technology, Xihua Normal University, and Xichang College. Data were obtained through a self-report questionnaire administered via Wenjuanxing, a widely used online survey platform in China. Only female undergraduate students aged 18–25 who met the inclusion criteria (Chinese nationality, enrolled as freshmen to senior undergraduates, able to understand the purpose of the study, and willing to voluntarily participate with informed consent) were included in the study. Participants who self-reported a prior diagnosis of a serious physical or mental health condition or an eating disorder, as well as those providing invalid responses (e.g., implausible out-of-range anthropometric values such as extremely high body weight), were excluded from the analysis.

### 2.2 Ethical clearance and permission

This study was approved by the Research Ethics Committee of the National University of Malaysia (No.: UKM PPI/111/8/JEP-2024-182) and the Academic Committee of Yibin University (No.: 20231008001) The researchers provided an explanation of the study protocol to participants, and informed consent detailing the purpose, risks, benefits, methods, and other ethical considerations was presented before the participants could answer the online questionnaire.

### 2.3 Measures

#### 2.3.1 Demographic data

College/university name, grade, major, age, ethnicity, height, weight, ideal weight, and household registration information were collected through the questionnaire. BMI (actual and ideal) was calculated as body mass (kg) divided by height squared (m^2^) from students’ self-reported height and weight/ideal weight. According to the Chinese standards, students’ BMIs were classified into four categories: underweight (<18.5 kg/m^2^), normal weight (18.5 ≥ BMI < 24 kg/m^2^), overweight (24.0 ≥ BMI < 28 kg/m^2^), and obese (≥28.0 kg/m^2^; National Health Commission of the People’s Republic of China, 2013).

#### 2.3.2 Body dissatisfaction

To evaluate BD, the present study employed the Three-dimensional Body Image Stimuli developed by Yan et al. (2021). This instrument consists of a set of eight three-dimensional female body figures, representing a continuum of body sizes ranging from extremely underweight to severely obese. Participants were asked to select two figures: one that they believed best represented their current body size (Current Body Size, CBS), and one that represented their ideal body size (Ideal Body Size, IBS). Based on these selections, BD scores were calculated using the formula BD = CBS–IBS. BD scores range from −7 to 7, with the absolute value of BD indicating the level of BD—the greater the absolute value, the higher the dissatisfaction. BD = 0 indicates satisfaction with one’s current body size; BD < 0 suggests a desire to be heavier, while BD > 0 score indicates a desire to be thinner (Yan et al., 2021). While previous psychometric validation focused on structural validity and reliability (e.g., weighted kappa, test–retest, and inter-rater reliability) rather than internal consistency of BD scores (Yan et al., 2021), the tool has been successfully applied in large community samples. For example, in a study of 316 young adults in Beijing and Guangzhou, BD scores showed meaningful associations with BMI and other body measures, supporting its utility in evaluating BD (Yan et al., 2022).

#### 2.3.3 Fatness concern

To evaluate fatness concern, the Fatness subscale from the Negative Physical Self-Scale was employed. The Negative Physical Self-Scale was developed by Chen et al. (2006) to measure the body perception of young Chinese people. The scale consists of five subscales, including general appearance, facial appearance, shortness, fatness, and thinness. In the present study, only the Fatness subscale was used to measure participants’ concerns related to body fat. This subscale consists of 11 items (e.g., “I am very distressed when I think about my weight”; “When I weigh myself, I feel depressed”). The Fatness subscale have demonstrated good reliability and validity in samples of Chinese university students (Gao et al., 2012). In this study, the Cronbach’s alpha coefficient of this scale was 0.91.

#### 2.3.4 Perceived sociocultural pressure scale

The perceived sociocultural pressure scale was developed by Stice and Agras (1998). The questionnaire includes 8 questions to evaluate the pressure level from parents, companions, media, etc., such as “I feel from the family and need to change my physical appearance.” “I feel from the media (such as TV, magazines, etc.) that I need to change the pressure of my physical appearance.” Through level 1–5 scores, the Chinese version of this scale has a high degree of trust (Jackson and Chen, 2008). The higher the average value, the greater the pressure on the appearance of an individual. In this study, the Cronbach’s alpha coefficient of this scale was 0.91.

#### 2.3.5 Body image acceptance and action questionnaire

The scale was used to measure an individual’s acceptance and action toward their body image. It consists of 12 items, each of which aims to assess the flexibility and acceptance level of an individual’s body image (Sandoz et al., 2013). Each item of the questionnaire is reverse-scored, and the questionnaire is associated with increased psychological flexibility, reduced BD, and fewer DEBs. The Chinese version of the body image acceptance and action questionnaire, which has sound psychometric properties, was used in this study (He et al., 2021). Cronbach’s alpha coefficient for this study was 0.94.

#### 2.3.6 Three-factor eating questionnaire

In this study, DEBs were assessed using the Chinese version of the Three-Factor Eating Questionnaire (TFEQ-R18V3; Zhang et al., 2016), a revised form of the TFEQ-R21 (Rosnah et al., 2013). This instrument evaluates three subscales: UE, EE, and CR. UE captures the inclination to consume more than usual due to a loss of control over eating; EE refers to overeating in response to negative emotional states, such as loneliness, sadness, or anxiety; and CR reflects the deliberate limitation of food intake to manage or reduce body weight. It consists of 18 items, each rated on a four-point Likert scale. Raw scores are converted to a standardized 0–100 scale using the formula: [(raw score-lowest possible raw score)/possible raw score range] × 100. Higher scores on each subscale reflect greater levels of the corresponding eating behavior. The revised Chinese version of the TFEQ-R18V3 has demonstrated good reliability among college students and is considered a valid tool for measuring DEBs (Zhang et al., 2016). Each subscale (UE, CR, EE) was categorized into two levels based on median cut-off points: “low” (0 ≤ score < median) and “high” (score ≥ median; Camilleri et al., 2014). The internal consistency of the questionnaire was high, with a Cronbach’s alpha of 0.94 overall (UE: *α* = 0.94; EE: α = 0.94; CR: α = 0.93).

### 2.4 Statistical analysis

This study constructed three models, each exploring the factors related to high UE (model 1), high EE (model 2), and high CR (model 3). Data were analyzed using IBM SPSS Statistics version 27 software. Descriptive statistics were first calculated for continuous variables, reported as means (M) and standard deviations (SD). For categorical variables, frequencies (n) and percentages (%) were computed. To explore the relationships between each predictor (e.g., age, college, major, grade, household registration, BMI, ideal BMI, perceived sociocultural pressures, BD, fatness concern, body image flexibility) and each high-risk DEB (including UE, EE, and CR), simple binary logistic regression was performed. Reference categories included “other ethnicity,” “Sichuan Tourism University,” “Science and Engineering,” “Freshman,” “Rural,” “Body satisfaction,” and “Unconcerned about fatness.” Subsequently, multiple binary logistic regression analyses were conducted, incorporating only those variables that demonstrated significant associations (*p* < 0.05) in the initial models, to assess their combined effects while controlling for other variables. The analyses provided odds ratios and 95% confidence intervals to estimate the strength of these associations, with statistical significance set at *p* < 0.05.

## 3 Results

A total of 2071 participants completed the online questionnaire. During data cleaning, a total of 346 (16.6%) cases were deleted upon review of the responses due to outlier data and not meeting the inclusion/exclusion criteria (e.g., being male). A total of 1727 participants, mean aged 19.73 (1.47) years, were retained for the final analyses. Table 1 details the participants’ demographic characteristics.

**Table 1:** Demographic characteristics of participants (*N* = 1727).

Variable	*n* (%) or Mean ± SD
Age	19.73 ± 1.47
BMI (kg/m^2^)	20.15 ± 2.58
Ideal BMI (kg/m^2^)	18.61 ± 1.60
University/College	
Chengdu College of Arts and Sciences	332(19.2)
China West Normal University	322 (18.6)
Sichuan Tourism University	356 (20.6)
Southwest University of Science and Technology	382 (22.1)
Xichang University	335 (19.4)
Major	
Humanities	964 (55.8)
Science and Engineering	354 (20.5)
Social Studies	409 (23.7)
Grade	
Freshman	691(40)
Sophomore	467 (27)
Junior	452 (26.2)
Senior	117 (6.8)
Ethnicity	
Han	1,556 (90.1)
Others	161 (9.9)
BMI category	
Underweight	468 (27.1)
Normal weight	1,094 (63.3)
Overweight	126 (7.3)
Obese	39 (2.3)
Household Registration	
Urban	511 (29.6)
Rural	1,216 (70.4)
Perceived sociocultural pressures	16.97 ± 6.59
Body image	1.19 ± 1.48
Satisfaction	281 (16.3)
Dissatisfaction (too thin)	194 (11.2)
Dissatisfaction (too fat)	1,252 (72.5)
Fatness concern	1.19 ± 0.82
Yes	346 (20)
No	1,381 (80)
Body image flexibility	33.22 ± 14.56
Disordered eating behaviors	
Uncontrolled eating (UE)	48.42 ± 23.80
High	909 (52.6)
None/Low	818 (47.1)
Emotional eating (EE)	41.58 ± 20.80
High	874 (50.6)
None/Low	853 (49.4)
Cognitive restraint (CR)	44.67 ± 25.58
High	1,030 (59.6)
None/Low	697 (404)

SD, Standard Deviation; BMI, body mass index.

Students were from Chengdu College of Arts and Sciences (*n* = 332; 19.2%), China West Normal University (*n* = 322; 18.6%), Sichuan Tourism University (*n* = 356; 20.6%), Southwest University of Science and Technology (*n* = 382; 22.1%), and Xichang University (*n* = 335; 19.4%). Nine hundred and sixty-four (55.8%) students majored in humanities; 354 (20.5%) students majored in science and engineering. A large majority, 1,556 (90.1%) students were of Han nationality, and 1,216 (70.4%) were from rural areas. In terms of level of study, 691 (40%) were freshmen, 467 (27%) were sophomores, and 452 (26.2%) were juniors. In addition, the mean BMI was 20.15 ± 2.58 kg/m^2^, while the mean ideal BMI was 18.61 ± 1.60 kg/m^2^. Among the students, only 126 (7.3%) had overweight and 39 (2.3%) had obesity; most of them (63.3%) had a weight within the normal. However, only 281 (16.3%) of the participants were satisfied with their body, while 1,252 (72.5%) of participants wished to be thinner. Three hundred and forty-six (20%) had concerns about fatness, 909 (52.6%) had high UE, 874 (50.6%) had high EE, and 1,030 (59.6%) had high CR eating behaviors.

Variables that were significant in the univariate logistic regression were entered into the multiple logistic regression model. Table 2 shows the factors that were associated with UE. The model was significant, Chi^2^ (12) = 185.64, *p* < 0.001. The Hosmer-Lemeshow test was non-significant, indicating good model fit. The predictor accounted for 13.6% of the variance in UE, Nagelkerke *R*^2^ = 0.136. Older age (a*OR*: 1.089, 95%*CI* [1.012, 1.172], *p* = 0.023), higher perceived sociocultural pressures (a*OR*: 1.033, 95%*CI* [1.011, 1.055], *p* = 0.003), lower level of body image flexibility (a*OR*: 0.980, 95%*CI* [0.970, 0.990], *p* < 0.001) had higher odds of UE. Compared with Sichuan Tourism University, China West Normal University (a*OR*: 1.401, 95% *CI* [1.013, 1.936], *p* = 0.041) had higher odds of UE. Compared with the unconcerned-about-fatness group, the had-fatness-concern group (a*OR*: 1.490, 95% *CI* [1.091, 2.035], *p* = 0.012) had higher odds of UE (See Table 2).

**Table 2:** Factors associated with uncontrolled eating behavior.

Variable	Simple logistic regression					Multiple logistic regression				
	β	c*OR*	95% *CI*		*p*	β	a*OR*	95% *CI*		*p*
Age	0.082	1.085	1.017	1.158	0.013	0.085	1.089	1.012	1.172	0.023
Ethnicity										
Han	−0.210	0.811	0.589	1.115	0.198					
Others		1.000								
College/university										
Chengdu College of Arts and Sciences	0.302	1.353	1.003	1.826	0.048	0.169	1.184	0.856	1.638	0.307
Southwest University of Science and Technology	0.179	1.196	0.895	1.596	0.226	0.181	1.198	0.881	1.630	0.250
Xichang university	0.428	1.534	1.136	2.071	0.005	0.310	1.364	0.976	1.907	0.069
China West Normal University	0.445	1.561	1.152	2.115	0.004	0.337	1.401	1.013	1.936	0.041
Sichuan Tourism University		1.000								
Major										
Humanities	−0.199	0.820	0.642	1.047	0.111					
Social Studies	0.005	1.005	0.755	1.339	0.970					
Science and Engineering		1.000								
Grade										
Sophomore	0.182	1.199	0.948	1.518	0.130					
Junior	0.219	1.244	0.981	1.579	0.072					
Senior	0.203	1.225	0.827	1.816	0.312					
Freshman		1.000								
Household registration										
Urban	−0.066	0.936	0.761	1.151	0.529					
Rural		1.000								
BMI	0.130	1.130	1.095	1.184	<0.001	0.059	1.060	0.988	1.139	0.106
Ideal BMI	0.060	1.062	1.000	1.127	0.049	-0.048	0.953	0.864	1.050	0.331
Perceived sociocultural pressures	0.083	1.086	1.069	1.103	<0.001	0.033	1.033	1.011	1.055	0.003
Body image										
Dissatisfaction (too fat)	0.803	2.233	1.712	2.911	<0.001	0.281	1.324	0.977	1.795	0.070
Dissatisfaction (too thin)	−0.056	0.945	0.648	1.379	0.771	-0.034	0.966	0.647	1.443	0.866
Satisfaction		1.000								
Fatness concern										
Yes	1.118	3.060	2.358	3.972	<0.001	0.399	1.490	1.091	2.035	0.012
No		1.000								
Body image flexibility	−0.041	0.959	0.952	0.967	<0.001	-0.020	0.980	0.970	0.990	<0.001

c*OR*, crude odds ratio; a*OR*, Adjusted odds ratio; *CI*, Confidence interval.

Table 3 shows the factors that were associated with EE. Due to multicollinearity issues, body image flexibility was not entered into the adjusted regression model. The final model was significant, Chi^2^ (13) = 163.58, *p* < 0.001. The Hosmer-Lemeshow test was non-significant, indicating good model fit. The predictor accounted for 12.0% of the variance in EE, Nagelkerke *R*^2^ = 0.120. EE was significantly associated with higher perceived sociocultural pressures (a*OR*: 1.069, 95% *CI* [1.049, 1.088], *p* < 0.001), perceiving themselves as being too fat (a*OR*: 1.497, 95% *CI* [1.116, 2.007], *p* = 0.007) and having higher fatness concern (a*OR*: 1.514, 95% *CI* [1.125, 2.038], *p* = 0.006; see Table 3).

**Table 3:** Factors associated with emotional eating behaviors.

Variable	Simple logistic regression					Multiple logistic regression (Model 2)				
	B	c*OR*	95% *CI*		*p*	B	a*OR*	95% *CI*		*p*
Age	0.092	1.096	1.028	1.160	0.005	0.140	1.150	0.991	1.334	0.066
Ethnicity										
Han	−0.090	0.914	0.666	0.666	0.577					
Others		1.000								
College/University										
Chengdu College of Arts and Sciences	0.297	1.346	0.997	1.818	0.052	0.152	1.164	0.840	1.612	0.361
Southwest University of Science and Technology	0.248	1.281	0.959	1.711	0.094	0.226	1.254	0.923	1.702	0.148
Xichang university	0.387	1.472	1.091	1.987	0.011	0.211	1.235	0.881	1.733	0.221
China West Normal University	0.399	1.490	1.101	2.018	0.010	0.260	1.297	0.937	1.795	0.117
Sichuan Tourism University		1.000					1.000			
Major										
Humanities	−0.123	0.885	0.693	1.129	0.325					
Social Studies	−0.038	0.963	0.724	1.280	0.793					
Science and Engineering		1.000								
Grade										
Sophomore	0.301	1.351	1.068	1.710	0.012	−0.040	0.961	0.608	1.519	0.865
Junior	0.248	1.282	1.011	1.626	0.040	−0.387	0.679	0.326	1.414	0.301
Senior	0.296	1.340	0.908	1.992	0.140	0.076	1.079	0.797	1.461	0.622
Freshman		1.000					1.000			
Household registration										
Urban	0.160	1.174	0.950	1.444	0.129					
Rural		1.000								
BMI	0.080	1.090	1.05	1.133	<0.001	−0.016	0.984	0.940	1.030	0.492
Ideal BMI	0.002	1.002	0.945	1.063	0.936					
Perceived sociocultural pressures	0.085	1.089	1.072	1.106	<0.001	0.066	1.069	1.049	1.088	<0.001
Body image										
Dissatisfaction (too fat)	0.717	2.049	1.571	2.672	<0.001	0.403	1.497	1.116	2.007	0.007
Dissatisfaction (too thin)	−0.108	0.898	0.614	1.313	0.898	−0.255	0.775	0.521	1.153	0.208
Satisfaction		1.000					1.000			
Fatness concern										
Yes	0.965	2.624	2.041	3.374	<0.001	0.415	1.514	1.125	2.038	0.006
No		1.000					1.000			
Body image flexibility^†^	−0.042	0.959	0.952	0.966	<0.001	-				

c*OR*, Crude odds ratio; a*OR*, Adjusted odds ratio; *CI*, Confidence interval. ^†^Not tested in the multiple logistic regression model due to multicollinearity issues.

Table 4 shows the factors that were associated with CR. Similar to the EE multiple logistic regression model, body image flexibility was not entered into the adjusted model due to multicollinearity issues. The model was significant, Chi^2^ (5) = 320.14, *p* < 0.001. The Hosmer-Lemeshow test was non-significant, indicating good model fit. The predictor accounted for 22.9% of the variance in CR, Nagelkerke *R*^2^ = 0.229. Higher perceived sociocultural pressures (a*OR*: 1.069, 95% *CI* [1.048, 1.090], *p* < 0.001) were associated with higher odds of CR. Compared with individuals satisfied with their body image, those who perceived themselves as too fat had significantly higher odds of CR (a*OR*: 2.160, 95% *CI* [1.608, 2.901], *p* < 0.001), while those who perceived themselves as too thin had significantly lower odds (a*OR*: 0.479, 95% *CI* [0.315, 0.729], *p* = 0.001). In addition, compared with those unconcerned about fatness, individuals with fatness concern exhibited higher odds of CR (a*OR*: 2.403, 95% *CI* [1.681, 3.437], *p* < 0.001; see Table 4).

**Table 4:** Factors associated with cognitive restraint behaviors.

Variable	Simple logistic regression					Multiple logistic regression (Model 3)				
	B	c*OR*	95% *CI*		*p*	B	a*OR*	95% *CI*		*p*
Age	0.016	1.016	0.952	1.085	0.628					
Ethnicity										
Han	0.053	1.055	0.765	1.453	0.744					
Others		1.000								
College/University										
Chengdu College of Arts and Sciences	0.091	1.095	0.807	1.487	0.560					
Southwest University of Science and Technology	−0.015	0.985	0.734	1.321	0.919					
Xichang university	0.006	1.006	0.742	1.362	0.972					
China West Normal University	0.002	1.002	0.737	1.362	0.990					
Sichuan Tourism University		1.000								
Major										
Humanities	0.095	1.099	0.858	1.408	0.453					
Social Studies	0.030	1.031	0.772	1.376	0.838					
Science and Engineering		1.000								
Grade										
Sophomore	0.092	1.096	0.862	1.393	0.454					
Junior	0.019	1.019	0.801	1.297	0.877					
Senior	0.195	1.215	0.810	1.822	0.346					
Freshman		1.000								
Household registration										
Urban	−0.020	0.980	0.794	1.209	0.850					
Rural		1.000								
BMI	0.216	1.241	1.186	1.298	<0.001	0.040	1.041	0.989	1.096	0.125
Ideal BMI	0.046	1.047	0.986	1.113	0.136					
Perceived sociocultural pressures	0.106	1.111	1.092	1.131	<0.001	0.067	1.069	1.048	1.090	<0.001
Body image										
Dissatisfaction (too fat)	1.208	3.348	2.564	4.373	<0.001	0.770	2.160	1.608	2.901	<0.001
Dissatisfaction (too thin)	−0.661	0.516	0.346	0.770	0.001	−0.736	0.479	0.315	0.729	0.001
Satisfaction		1.000					1.000			
Fatness concern										
Yes	1.652	5.218	3.798	7.170	<0.001	0.877	2.403	1.681	3.437	<0.001
No		1.000					1.000			
Body image flexibility^†^	−0.078	0.925	0.917	0.934	<0.001	-				

c*OR*, Crude odds ratio; a*OR*, Adjusted odds ratio; *CI*, Confidence interval. ^†^Not tested in the multiple logistic regression model due to multicollinearity issues.

## 4 Discussion

Our findings revealed that a significant proportion of participants exhibited high levels of DEBs, with over half reporting UE, EE, and CR. The prevalence rates observed in our study were consistent with those found in similar populations (Jiménez Limas et al., 2022; Yong et al., 2021), suggesting that DEBs were a pervasive issue among these students. However, our results also highlighted the need for a more nuanced understanding of the specific factors that were associated with different types of DEBs.

The results of the multiple logistic regression in model 1 indicated that older age was significantly associated with higher odds of UE among female college students. This might be partly explained by age-related physiological changes and increasing challenges in weight management, which can heighten food cravings and reduce the ability to resist temptation. Moreover, students, particularly older ones, might also engage in restricting food intake as a strategy to control body weight, which could lead to food cravings and disinhibition (i.e., the loss of control over eating behavior; Coulthard et al., 2021). Previous studies have indicated that, with increasing age, the association between higher body weight and greater levels of UE among females may become more pronounced (Abdella et al., 2019). Moreover, individuals aged ≤ 25 have been found to report stronger cravings for carbohydrates and fats, along with higher scores in emotional and UE (Anglé et al., 2009). The results also revealed that, compared with Sichuan Tourism University, students from China West Normal University had significantly higher odds of UE. This disparity may reflect regional economic and educational inequalities, which limit health literacy and self-regulatory behaviors regarding healthy eating (Lu et al., 2026; Sun et al., 2025).

The results of this study indicated that higher perceived sociocultural pressures were significantly associated with UE in model 1. Consistent with prior findings, perceived sociocultural pressures, especially pressures from family, peers, and the media, have been identified as significant predictors of DEBs among female university students (Fu et al., 2022; Johnson et al., 2015). For instance, a cross-sectional study conducted among Chinese university students revealed that perceived stress had a direct impact on binge eating behavior (Li et al., 2024). The findings in model 2 further confirmed the significant association between perceived sociocultural pressures with EE. Previous studies have shown that perceived stress could more likely trigger the desire to eat, leading to the consumption of more high-fat, high-calorie foods (Chen, 2023; Godet et al., 2022). Individuals may turn to food as a coping mechanism for negative emotions. Research has indicated that such emotional distress can trigger eating in the absence of physical hunger as a way to obtain psychological comfort (White et al., 2024). For instance, a study on college students showed that perceived stress was positively correlated with EE and negatively correlated with dietary self-regulation (Ling and Zahry, 2021). Model 3 further showed that higher perceived sociocultural pressures were also significantly associated with CR, suggesting that women under stronger appearance-related pressures may attempt to regulate weight/shape through greater dietary restriction (Stice and Van Ryzin, 2019). However, CR can be a double-edged strategy: when restraint is prolonged, rigid, or anxiety-driven, it may increase vulnerability to rebound overeating and emotional distress, thereby exacerbating UE and EE (Duarte et al., 2020; Natacci and Ferreira Júnior, 2011; Rossi et al., 2023). Consistent with this view, evidence indicates that CR may paradoxically predict binge-eating tendencies and weight gain over time in some individuals, while a more flexible form of restraint may relate to more adaptive outcomes (Alqahtani and Alhazmi, 2025).

Meanwhile, the results showed that lower body image flexibility significantly predicted higher levels of UE in model 1. While body image flexibility could not be tested in the multiple logistic regression models due to multicollinearity issues, they were highly significant (*p* < 0.001) in the simple logistic regression analyses for both EE and CR. Body image flexibility refers to the psychological flexibility exhibited in situations of EDs and BD (Sandoz et al., 2013). Previous studies have shown that body image flexibility could help individuals maintain a more flexible and adaptive attitude when facing negative body image, reducing UE caused by BD (Tan et al., 2019). This indicated that improving body image flexibility is meaningful for preventing and reducing BD and UE, and further research can be conducted. This study also found that body image flexibility was associated with EE in the univariate analysis, suggesting that improving body image flexibility might help reduce EE behaviors. Previous research has shown that negative memories and emotional experiences related to body image may exacerbate individuals’ difficulties in controlling eating behavior and managing weight, whereas body image flexibility may serve as a self-regulatory mechanism that helps buffer these effects (Duarte and Pinto-Gouveia, 2016). At the same time, the study revealed that body image flexibility was correlated with CR in the univariate analysis. Previous research indicated that body image flexibility was negatively correlated with eating and body image disturbances and general psychopathological constructs, while positively correlated with positive psychological constructs (Moore et al., 2014). These results suggested that body image flexibility could act as a protective factor, helping to reduce the occurrence of CR eating behaviors.

In model 3, the findings of this study were consistent with previous literature, indicating that factors such as BD and fatness concern were significantly associated with CR among female undergraduates. Among these, dissatisfaction with one’s body (particularly the perception of being too fat) and concern about being fat had become risk factors for the occurrence of restrictive eating, a point that had been confirmed in several studies (MacLeod et al., 2020; Yong et al., 2021). For instance, a study on college students found that BD was positively correlated with restrictive dieting scores. This meant that the higher the level of BD among college students, the higher their restrictive dieting scores (Hao et al., 2022).

### 4.1 Limitations

This study has several limitations. Firstly, the sample only includes Chinese female college students, which means we cannot infer gender differences, and the research conclusions may not be applicable to other specific groups or people from different cultural backgrounds. Future research could consider collecting data from different groups (e.g., male college students and adolescents) and across different cultures to further test the robustness of our findings. Secondly, this study is cross-sectional, which limits our ability to infer causal relationships. Future research would benefit from a longitudinal design. Thirdly, the dichotomization of certain variables in our analysis may have reduced statistical power and inflated errors, leading to potential biases in the results. This method should be reconsidered in future studies to better capture variability in the data and improve statistical accuracy. In addition, body image flexibility could not be tested in the multiple logistic regression models for EE and CR due to multicollinearity and had contributed to poor model fit. Next, this study relied on self-report questionnaires, which carry the risk of underreporting or overreporting. Participants may have underreported or overreported information in an attempt to present themselves in a more positive light. Finally, socioeconomic differences between the institutions included in the study were not fully explored, and these factors may have influenced the outcomes. Future research could examine how socioeconomic level (or other relevant differences) across institutions impacts the results and whether these differences contribute to variations in DEBs.

## 5 Conclusion

The present study indicates that UE, EE, and CR are prevalent among Chinese female undergraduates. Importantly, the correlates varied across DEB types: UE was linked to older age, stronger perceived sociocultural pressures, lower body image flexibility, university context, and greater fatness concern, whereas EE and CR were primarily associated with sociocultural pressures, fatness concern, and BD. Weight self-perception also showed distinct associations, with perceiving oneself as “too fat” increasing the likelihood of EE and CR, while perceiving oneself as “too thin” was associated with lower odds of CR.

These findings suggest that interventions should be tailored to specific DEB patterns rather than adopting a single approach. Efforts that reduce sociocultural appearance pressures and address fatness concern and BD may be broadly beneficial, while enhancing body image flexibility may be particularly important for preventing UE. Future research should further explore the pathways linking these factors to different forms of DEBs.

## Data Availability

The original contributions presented in the study are included in the article/supplementary material, further inquiries can be directed to the corresponding authors.
